# Post-Flood Impacts on Occurrence and Distribution of Mycotoxin-Producing Aspergilli from the Sections *Circumdati*, *Flavi,* and *Nigri* in Indoor Environment

**DOI:** 10.3390/jof6040282

**Published:** 2020-11-12

**Authors:** Daniela Jakšić, Miranda Sertić, Sándor Kocsubé, Ivana Kovačević, Domagoj Kifer, Ana Mornar, Biljana Nigović, Maja Šegvić Klarić

**Affiliations:** 1 Department of Microbiology, Faculty of Pharmacy and Biochemistry, University of Zagreb, 10000 Zagreb, Croatia; msegvic@pharma.hr; 2 Department of Pharmaceutical Analysis, Faculty of Pharmacy and Biochemistry, University of Zagreb, 10000 Zagreb, Croatia; msertic@pharma.hr (M.S.); amornar@pharma.hr (A.M.); bnigovic@pharma.hr (B.N.); 3 Department of Microbiology, Faculty of Science and Informatics, University of Szeged, H-6726 Szeged, Közép fasor 52, Hungary; shigsanyi@gmail.com; 4 Faculty of Pharmacy and Biochemistry, University of Zagreb, 10000 Zagreb, Croatia; ivana.kovacevic444@gmail.com; 5 Department of Biophysics, Faculty of Pharmacy and Biochemistry, University of Zagreb, 10000 Zagreb, Croatia; dkifer@pharma.hr

**Keywords:** Aspergilli, *Circumdati*, *Flavi*, *Nigri*, post-flood, indoor fungi

## Abstract

Mycotoxin-producing Aspergilli (*Circumdati*, *Flavi,* and *Nigri*), usually associated with contaminated food, may also cause respiratory disorders and are insufficiently studied in water-damaged indoor environments. Airborne (*N* = 71) and dust borne (*N* = 76) Aspergilli collected at post-flood and control locations in Croatia resulted in eleven different species based on their calmodulin marker: *A. ochraceus*, *A. ostianus*, *A. pallidofulvus, A. sclerotiorum,* and *A. westerdijkiae* (*Circumdati*); *A. flavus* (*Flavi*); and *A. tubingensis*, *A. welwitschiae*, *A. niger*, *A. piperis,* and *A. uvarum* (*Nigri*). Most of the airborne (73%) and dust borne (54%) isolates were found at post-flood locations, and the highest concentrations measured in indoor air (5720 colony-forming units (CFU)/m^3^) and dust (2.5 × 10^5^ CFU/g) were up to twenty times higher than in the control locations. *A. flavus* dominated among airborne isolates (25%) at the unrepaired locations, while 56% of the dust borne Aspergilli were identified as *A. tubingensis* and *A. welwitschiae*. The ability of identified isolates to produce mycotoxins aflatoxin B_1_ (AFB_1_), fumonisin B_2_ (FB_2_), and ochratoxin A were assessed by LC-MS analysis. All ochratoxin A (OTA)-producing *Circumdati* belonged to *A. westerdijkiae* (13.7 ± 15.81 µg/mL); in the section, *Flavi* *A. flavus* produced AFB_1_ (2.51 ± 5.31 µg/mL), while *A. welwitschiae* and *A. niger* (section *Nigri*) produced FB_2_ (6.76 ± 13.51 µg/mL and 11.24 ± 18.30 µg/mL, respectively). Water damage dominantly supported the occurrence of aflatoxigenic *A. flavus* in indoor environments. Yet unresolved, the causal relationship of exposure to indoor Aspergilli and adverse health effects may support the significance of this research.

## 1. Introduction

As a result of climate change, we are witnessing an increasing number of storms, hurricanes, and other events resulting in flooding. Among the numerous consequences associated with these disastrous events, fungal proliferation complicates and increases the costs of renovations, and it represents a health threat to those exposed to it [1,2,3,4,5]. Building dampness or moisture problems are a typical cause of “building decay”, primarily because water promotes fungal growth, and fungi degrade the substrate on which they grow. With an ability to tolerate water activities (a_w_) 0.75–0.90, Aspergilli are the most common indoor fungi [6], while at the same time, their concentration in outdoor air is low. Aspergilli from the section *Nidulantes* series *Versicolores* are the most prevalent indoor Aspergilli [7,8,9,10,11,12,13,14,15], while only a limited number of studies reported Aspergilli from the sections *Circumdati, Flavi,* and *Nigri* in air or dust samples from indoor environments [16,17,18,19,20,21,22,23,24,25]. The *Aspergillus* species belonging to the sections *Circumdati, Flavi,* and *Nigri* are primary colonizers that can grow at a lower water activity than secondary (e.g., *Cladosporium* spp.) and tertiary colonizers (e.g., *Trichoderma* spp., *Stachybotrys* spp., and *Chaetomium* spp.). In addition, they are among the microbes most resistant to various disinfecting compounds [26]. Thus, they may remain in the indoor environment following water damage even after certain remediation activities are applied e.g., removing excess water, drying, ventilation, disinfection, etc. Unless thorough sporicidal fumigation is performed, their spores may remain indoors and proliferate when favorable conditions occur. Therefore, they could be a good indicator of successful or non-successful indoor post-flood remediation.

There are many reasons why monitoring Aspergilli from the sections *Circumdati, Flavi,* and *Nigri* matters. They may cause a variety of clinical pictures in humans, from simple allergies to severe asthma with fungal sensitization, especially in children [27]. They are also implicated in severe illnesses such as chronic pulmonary aspergillosis [28,29], and there are reports of its association with exposure to moulds at home [30]. A special concern arises from the ability of these species to produce mycotoxins such as ochratoxin A (OTA), aflatoxin B_1_ (AFB_1_), and fumonisin B_2_ (FB_2_), which can be deposited in various substrates, including dust in an indoor environment. In addition to their toxic, mutagenic, genotoxic, and cancerogenic properties [31,32,33,34,35], all of these mycotoxins may modulate immune responses and, as such, they may contribute to impaired response to infection or to chronic inflammatory disorders [36].

In 2014, catastrophic floods occurred in the areas of east Croatia, northwest Bosnia, and central Serbia [37]. The municipality of Gunja, in eastern Croatia, suffered heavy damage. The water levels measured in housing ranged from a few cm to 4 m, and the majority of inhabitants were evacuated. Two years after this catastrophic flood, most of these houses have undergone repair. While fungal indoor colonisation was still visible in some of the repaired homes, unrepaired houses were heavily contaminated by fungal growth. In order to evaluate post-flood fungal damage related to the presence of Aspergilli, air samples and dust were collected at repaired and unrepaired locations in Gunja during the winter and summer of 2016 and 2017. Houses as well as the school in Gornji Stupnik were chosen as the control locations as they are settled in a similar riverside environment in western Croatia. Airborne and dust borne AFB_1_, OTA, and FB_2_ producing and non-producing Aspergilli from the sections *Circumdati*, *Flavi,* and *Nigri* were studied by cultivation on specific agar plates and identified to species level based on partial calmodulin (*CaM*) sequences. To assess mycotoxin-producing abilities, a micro-extraction procedure [38,39] was applied for an each isolate following liquid chromatography-tandem mass spectrometry (LC/MS) analysis of mycotoxins AFB_1_, OTA, and FB_2_ in corresponding mould extracts.

## 2. Materials and Methods

### 2.1. Solvents, Reagents, and Mycotoxin Standards

Czapek Yeast Extract Agar (CYA) was prepared from Czapek Dox Broth (Difco; Becton, Dickinson and Company, Franklin Lakes, NJ, USA), Yeast Extract (Difco; Becton, Dickinson and Company, Franklin Lakes, NJ, USA), trace elements salts ZnSO_4_ × 7 H_2_O and CuSO_4_ × 5 H_2_O, and agar–agar (Kemig, Zagreb, Croatia) following the manual [40]. Malt Extract Agar (MEA), Dichloran 18%-glycerol agar (DG-18), and Dichloran Rose-Bengal Cloramphenicol Agar (DRBC) were purchased from Oxoid Limited, Thermo Fisher Scientific, Basingstoke, UK. Distilled water was prepared with Niro-VV-Atlantic 3 (Nirosta d. o. o., Zagreb, Croatia). Molecular biology grade water was purchased from Lonza (Basel, Switzerland). The solvents acetonitrile (MeCN) and methanol (MeOH) were of LC-MS grade and purchased from Kemig (Zagreb, Croatia). Dichloromethane, ethyl acetate (EtAc), formic acid (HCOOH), and acetic acid (AcOH) were of pro analysi grade and purchased from Lach-Ner d.o.o. Zagreb, Croatia. Aflatoxin B_1_ (AFB_1_) and ochratoxin A (OTA) were purchased from Sigma-Aldrich (Deisenhofen, Germany) and fumonisin B_2_ (FB_2_) was purchased from Cayman Chemical Company (Ann Arbor, MI, USA).

### 2.2. Sampling: Source, Locations, and Periods

According to the information obtained through interviews with the residents of Gunja, the sampling locations were selected based on renovation activities following the flood. When the water receded after the flood, excess water was pumped out of the buildings, and the entire area was treated with disinfectant fluids. Since then, the majority of the homes in the village have undergone thorough renovation, including drying, reconstruction works, wall painting and repeated application of chlorine-based disinfectant (Izosan^®^ G). Among these thoroughly repaired locations, we chose five houses and the elementary school as post-flood repaired locations, and five houses were selected from those that had not undergone thorough remediation (unrepaired locations). In addition, six control locations were included in this study, which were comprised of five houses and one elementary school from a similar riverside area that was not affected by flooding. Sampling of air and dust was performed in four sampling periods, late winter (February) and late summer (September) of 2016 and 2017. Altogether, 60 indoor and 12 outdoor air samples, along with 12 samples of dust (control and repaired locations), and 40 indoor and 10 outdoor air samples, along with 10 samples of dust from unrepaired locations, were taken in each sampling period.

Airborne moulds were sampled 1 m above ground using a Mas 100 Eco air sampler (Merck, Darmstadt, Germany) with 400 holes (hole to agar impactor) and DG-18 and MEA plates [41]. A volume of 50 L was sampled with the impaction velocity of the sampler at approximately 10.8 m/s and an airflow rate of 100 L/min. The dust samples were collected by occupants in a nylon sampling sock by vacuuming an area of 1–3 m^2^ in home/school locations. After field sampling, a mycological analysis of dust samples was performed using the plate count method; 1 g of dust was suspended in peptone broth (9 mL) and dilutions from 10^−1^ to 10^−5^ were plated (0.1 mL) on DG-18 and DRBC [41]. The plates with prepared air and dust samples were incubated for five days in the dark at 25 ± 2 °C. After the mould colonies were developed, in order to separate Aspergilli from the sections *Circumdati*, *Flavi,* and *Nigri*, Aspergilli were re-isolated on CYA and incubated for seven days in the dark at 25 ± 2 °C.

#### Statistical Analysis

The number of colony-forming units (CFU) of total Aspergilli and each *Aspergillus* section, per m^3^ of air or per gram of dust, was analysed by mixed modelling. As the fixed factors group was nested within the location (levels Gunja: Unrepaired locations, Repaired locations, Outdoor air–air only, and Gornji Stupnik: Control locations, Outdoor air–air only) crossed with Season (levels Winter and Summer). The object identifier was included as a random intercept to account for multiple measures taken from the same object. Due to skewed distribution, CFU/m^3^ or CFU/g were transformed by inverse transformation of ranks to the standard normal distribution prior to the analysis. Based on the fitted model, *t*-test post-hoc was applied for Winter and Summer periods separately, comparing the difference in CFU/m^3^ or CFU/g between Unrepaired and Repaired locations, between Repaired locations and Control locations, and between Outdoor air in Gunja and Gornji Stupnik (air models only). Due to multiple testing, the false discovery rate was controlled using the Bonferroni method. Statistical significance was set at a level of 0.05. All statistical analyses were performed in R.

### 2.3. Identification of Aspergilli to the Species Level

The extraction of template DNA for PCR amplification was achieved using a NucleoSpin® Plant II kit (Macherey-Nagel GmbH & Co. KG, Düren, Germany), which was optimised and modified for the extraction of genomic DNA from fungi. The mycelia of three-day-old Aspergilli isolates cultured on CYA in darkness at 25 °C were scrapped by a sterile loop and transferred to 1 mL of SDS-based buffer in a sterile falcone tube containing approximately 1 g of sterile glass beads and vortexed at maximum speed for 3–5 min. Protein-precipitation reagent (500 µL) was added to the mixture, vortexed for 30 s, and incubated for 10 min at −20 °C. After this, the protocol provided by the manufacturer was followed. The isolated genomic DNA was used as the template DNA for PCR reactions. Part of the calmodulin gene (*CaM*) was amplified with 0.2 μM cmd5 and cmd6 primers (Hong et al., 2006). The reaction mixture, set to 20 μL volume, contained Hot Start polymerase Master Mix 2x and water (Takara Bio Inc, Kusatsu, Japan). Amplifications (T-100 Thermal Cycler, Bio Rad, Hercules, USA) in 35 cycles: 95 °C (5 min 1st, then 20 s), 56 °C (20 s), 72 °C (40 s; 5 min final). The successful amplifications were confirmed by electrophoresis (Sub-Cell® GT Agarose Gel Electrophoresis Systems, BioRad, Hercules, USA) on a 1.5% agarose gel by mixing the PCR product with fluorescent dye GelStar (Basel, Switzerland) against the reference ladder (100 bp, Lonza, Basel, Switzerland) under UV light (UV transilluminator Uvitec, Cambridge, UK). NucleoSpin® Gel and PCR Clean-up (Macherey-Nagel GmbH & Co., Düren, Germany) was used for purification of the PCR products following the procedure provided by manufacturer. The sequencing was done using the same pair of primers at Macrogen Inc., Amsterdam, The Netherlands.

#### Phylogeny

Sequences of the ex-type strains of all the species in section *Circumdati*, *Flavi,* and *Nigri* were obtained from the most recent phylogeny study of *Aspergillus* [42], downloaded from GenBank, and added to the analyses. An alignment of partial *CaM* sequences was made using Prank v 0.150803 [43] with default settings. The datasets were partitioned to exons and introns, and the best-fitting model was estimated by using ModelTest-NG v. 0.1.4 [44], allowing only gamma rate heterogeneity. Model selection was based on the corrected Akaike Information Criterion [45,46]. The proposed substitution models for intron and exon partitions, respectively, were HKY+G4 and TrN+G4 (section *Circumdati*), GTR+G4 and TIM1+G4 (section *Flavi*), and HKY+G4 and TIM2+G4 (section *Nigri*). Phylogenetic reconstruction was conducted using Maximum Likelihood (ML) analyses using RAxML-NG v0.9.0 [47]. Branch support was estimated by 500 bootstrap replicates. All identified isolates were stored under the appropriate number in the working microbial culture collection of the Department of Microbiology, Faculty of Pharmacy and Biochemistry, University of Zagreb.

### 2.4. Mycotoxin-Producing Abilities

Each of the *Aspergillus* isolates was extracted as described previously [19] following the procedure by Smedsgaard [38] for isolates from the sections *Flavi* and *Circumdati*, i.e., detection of AFB_1_ and OTA, and the procedure by Frisvad et al. [39] for the section *Nigri,* i.e., detection of FB_2_. The extracts were ultrasonicated, and the organic phases were separated, filtered through polypropylene (PP) or polytetrafluoroethylene (PTFE) syringe filters, evaporated to dryness in rota-vapour concentrator (Concentrator Plus, Eppendorf, Germany), weighed, dissolved in MeOH:H_2_O 0.7:0.3, *v/v*, and stored at −20 °C until the LC/MS analysis. All experiments were performed using the Agilent 1100 Series LC-MSD Trap system (Agilent Technologies, Waldbronn, Germany). Instrument control, data acquisition, and evaluation were done using ChemStation for LC 3D and LC/MSD Trap v. 5.2 software. The details about LC/MS parameters are included in Appendix A.

## 3. Results

### 3.1. The Airborne Aspergilli

**Figure 1:**
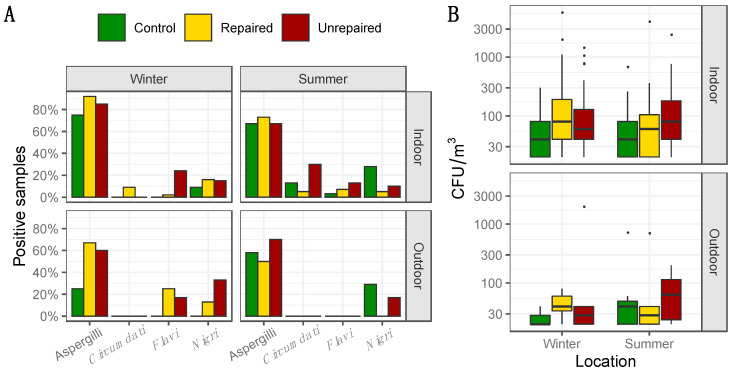
Percentage of positive samples where total airborne Aspergilli, as well as Aspergilli from the sections *Circumdati*, *Flavi,* and *Nigri,* were detected based on analysis of 60 indoor air and 12 outdoor air samples for control and repaired locations and 40 indoor and 10 outdoor air samples for unrepaired locations in each sampling period i.e., winter and summer (**panel A**); Concentration of total Aspergilli (colony-forming units (CFU)/m^3^) detected in indoor and outdoor air. Each location is represented by a box–whisker plot in a different colour in each sampling period (winter and summer) as specified (**panel B**).

The frequency and the concentrations of total Aspergilli in indoor and outdoor air samples from the post-flood locations (repaired and unrepaired) and control locations, including the proportion of Aspergilli from the sections *Circumdati*, *Flavi,* and *Nigri,* are presented in Figure 1, while the details regarding the concentrations of Aspergilli assigned to the sections *Circumdati*, *Flavi,* and *Nigri* are provided in the supplementary materials (Tables S1–S3). Aspergilli were dominantly isolated from indoor air collected at the repaired and the unrepaired locations in winter (92 and 85%, respectively), while they were less frequent in indoor air samples collected at the control locations (75%). In both sampling periods, the concentrations of Aspergilli in indoor air were higher than at the control locations, while the difference was less pronounced in the summer than in winter (Figure 1). The highest concentrations of Aspergilli in outdoor air were measured at the unrepaired locations, and they were twenty-five times higher than in outdoor air from the repaired locations and almost fifty times higher than in outdoor air from control locations (Figure 1). The opposite seasonal pattern was observed at the unrepaired locations compared to the control and repaired post-flood locations as their concentrations in outdoor air were ten times lower in the summer than in the winter sampling period.

When compared to other sections, Aspergilli from the section *Circumdati* were rare, isolated only from indoor air samples, and dominant at the post-flood locations (Figure 1). Statistically, there were significantly more Aspergilli from the section *Circumdati* in the indoor air of post-flood unrepaired locations compared to the repaired locations in Gunja in the summer sampling period (Supplementary Materials, Table S1), while in the winter period, there were significantly more Aspergilli from the section *Flavi* in the indoor air of unrepaired locations compared to the repaired locations in Gunja (Supplementary Materials, Table S2). In addition, they were dominant in outdoor air collected at the post-flood locations in winter, comprising 25% and 17% of total airborne Aspergilli in the samples from the repaired and unrepaired locations, respectively (Figure 1). Aspergilli from the section *Nigri* were more abundant at repaired locations in winter (Max 1100 CFU/m^3^). In the summer, this was inversed, as the black Aspergilli were up to five times more common in the samples from the control locations compared to the post-flood locations (Figure 1 and Supplementary Materials Table S3). Exceptionally high concentrations of black Aspergilli were observed in the outdoor air samples collected at the unrepaired locations (Max 1980 CFU/m^3^) in winter. In the summer, at both post-flood locations, the concentrations of black Aspergilli in indoor and outdoor air decreased (Supplementary materials, Table S3).

### 3.2. The Dust Borne Aspergilli

**Figure 2:**
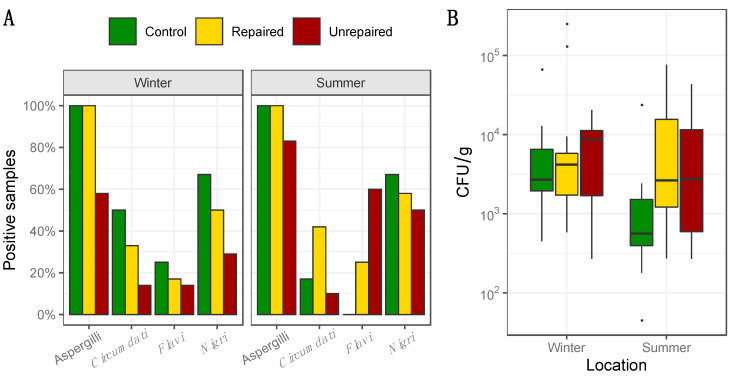
Percentage of positive samples where total dust borne Aspergilli, as well as Aspergilli from the sections *Circumdati*, *Flavi,* and *Nigri,* were detected based on analysis of 12 samples of dust collected at control and repaired locations, and 10 samples of dust collected at unrepaired locations in each sampling period, i.e., winter and summer (**panel A**); Concentration of total Aspergilli (CFU/g) detected in dust. Each location is represented by a box–whisker plot in a different shade of gray in each sampling period (winter and summer) as specified (**panel B**).

The frequency and concentrations of total Aspergilli in dust collected at the post-flood locations (repaired and unrepaired) and control locations, including the proportion of Aspergilli from the sections *Circumdati*, *Flavi,* and *Nigri,* are presented in Figure 2. Details regarding the concentrations of Aspergilli assigned to the sections *Circumdati*, *Flavi,* and *Nigri* are provided in the Supplementary Materials (Tables S4–S6). The dust borne Aspergilli were present in every dust sample collected at the control locations and repaired post-flood locations. The highest concentrations of total dust borne Aspergilli were measured in the dust from the repaired locations in the winter sampling period (Max 2.5 × 10^5^ CFU/g). They were up to four times higher than in the dust from the control locations and up to ten times higher than in the dust from the unrepaired locations (Figure 2). At both locations, concentrations of the dust borne Aspergilli declined in the summer sampling period (Figure 2). At the unrepaired locations, the dust borne Aspergilli were less frequent and presented in lower concentrations compared to other locations. In addition, the opposite seasonal pattern was observed as the concentrations of the dust borne Aspergilli measured in the summer doubled compared to the winter sampling period (Figure 2).

While Aspergilli from the section *Circumdati* were not present in indoor or outdoor air at the control locations in the winter sampling period, their maximum concentration in dust samples (42,727 CFU/g) were up to seventy times higher compared to other locations (Supplementary Materials, Table S4). However, their concentrations drastically dropped in the summer (max 50 CFU/g). At post-flood locations, the dust borne *Circumdati* were more frequent at repaired compared to unrepaired locations, where they were detected in only one sample (Figure 2, Supplementary Materials, Table S4). Aspergilli from the section *Flavi* dominated in dust collected at the post-flood locations, while at the control locations, they were obtained only from winter samples (Figure 2). Their maximum concentrations in the dust collected at the repaired locations in winter (545 CFU/g) were more than three times higher compared to the control locations. The highest concentrations of *Flavi* were measured in dust from unrepaired locations in the summer (17,568 CFU/g), and they were eighty-seven times higher than at the same location in winter and thirty-five times higher than in dust from repaired post-flood locations in the same sampling period (Supplementary Materials, Table S5). Aspergilli from the section *Nigri* comprised 50–67% of the dust borne Aspergilli at control and post-flood repaired locations. The highest concentrations of dust borne *Nigri* were measured in the samples collected in the winter sampling period at the control locations (4545 CFU/g). Their median concentration (1024 CFU/g) was 1.5 times higher than in the samples from the repaired locations. Summer concentrations of black Aspergilli in dust from the control locations were about ten times lower than in the winter period, while at repaired locations, they were similar in both winter and summer (Supplementary Materials, Table S6). Black Aspergilli followed the previously mentioned opposite seasonal pattern at the unrepaired locations, as they prevailed in summer compared to winter (Supplementary Materials, Table S6). However, their median concentration in dust from unrepaired locations (143 CFU/g) was 3.6 times lower compared to the repaired locations (Supplementary Materials, Table S6).

### 3.3. Identification of the Species from the Sections Circumdati, Flavi, and Nigri and Mycotoxin-Producing Capacities

Airborne (N = 71) and dust borne (N = 76) Aspergillus isolates were identified as eleven different species: *A. ochraceus*, *A. ostianus*, *A. pallidofulvus*, *A. sclerotiorum*, and *A. westerdijkiae* (section *Circumdati*), *A. flavus* (section *Flavi*), *A. niger*, *A. piperis*, *A. tubingensis*, *A. uvarum*, and *A. welwitschiae* (section *Nigri*). The identification was based on the comparison of the DNA sequence of a PCR-amplified *CaM* fragment from each isolate to the *CaM* sequences for reference strains and supported by phylogenetic analysis (Supplementary materials, Figures S1–S3). The extracts prepared from each of the isolates were tested for the presence of the mycotoxin relevant for each section, i.e., ochratoxin A (OTA) in the section *Circumdati*, aflatoxin B_1_ (AFB_1_) in the section *Flavi*, and fumonisin B_2_ (FB_2_) in the section *Nigri* (Table 1, Supplementary Materials, Tables S7–S9). Additionally, the extracts prepared from the isolates of *A. niger* and *A. welwitschiae* from the section *Nigri* were tested on OTA, but it was not detected. The HPLC-ESI-MS chromatograms are shown for a representative isolate producing OTA, AFB_1_, and FB_2_, along with the corresponding standards (OTA, AFB_1_, and FB_2_) in the Supplementary Materials, Figures S4–S6. 

All OTA-producing and AFB_1_-producing isolates belonged to *A. westerdijkiae* and *A. flavus,* respectively, while FB_2_-producing isolates belonged to *A. niger* and *A. welwitschiae* (Table 1, Supplementary Materials, Tables S7–S9).

The OTA-producing airborne isolates were from indoor air collected at the repaired locations in winter and at the control locations in the summer sampling period. The only dust borne isolate was from the dust collected at the repaired locations in the summer (Table 1, Supplementary Materials, Table S7).

The AFB_1_-producing *A. flavus* isolates were only detected among the isolates from the post-flood locations (Table 1, Supplementary Materials, Table S8). Only one AFB_1_-producing airborne isolate originated from the repaired locations, and it was isolated from outdoor air collected in winter. All other isolates of AFB_1_-producing *A. flavus* were winter airborne isolates and summer dust borne isolates from the unrepaired locations (Table 1, Supplementary Materials, Table S8).

FB_2_-producing *A. niger* isolates were recovered from an indoor and an outdoor air sample, respectively, collected at the control locations in the summer. A single dust borne isolate of FB_2_-producing *A. niger* was isolated at a repaired location in winter (Table 1, Supplementary Materials, Table S9). Both airborne isolates of FB_2_-producing *A. welwitschiae* were recovered from indoor air collected at the repaired locations in winter and summer, respectively. The dust borne isolates of FB_2_-producing *A. welwitschiae* were collected at the control locations in both sampling periods, while in dust from both post-flood locations, they were isolated only in summer (Table 1, Supplementary Materials, Table S9).

### 3.4. Seasonal Distribution of the Species from the Sections Circumdati, Flavi, and Nigri at Post-Flood and Control Locations

**Figure 3:**
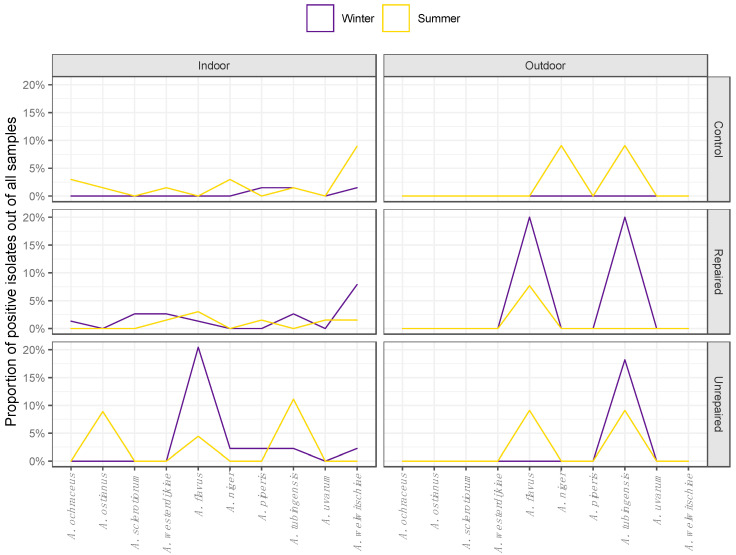
Seasonal distribution patterns of Aspergilli from the sections Circumdati (A. ochraceus, A. ostianus, A. sclerotiorum, and A. westerdijkiae), Flavi (A. flavus) and Nigri (A. niger, A. piperis, A. tubingensis, A. uvarum, and A. welwitschiae) in indoor and outdoor air during winter and summer sampling periods in repaired houses, unrepaired houses, and control houses.

**Figure 4:**
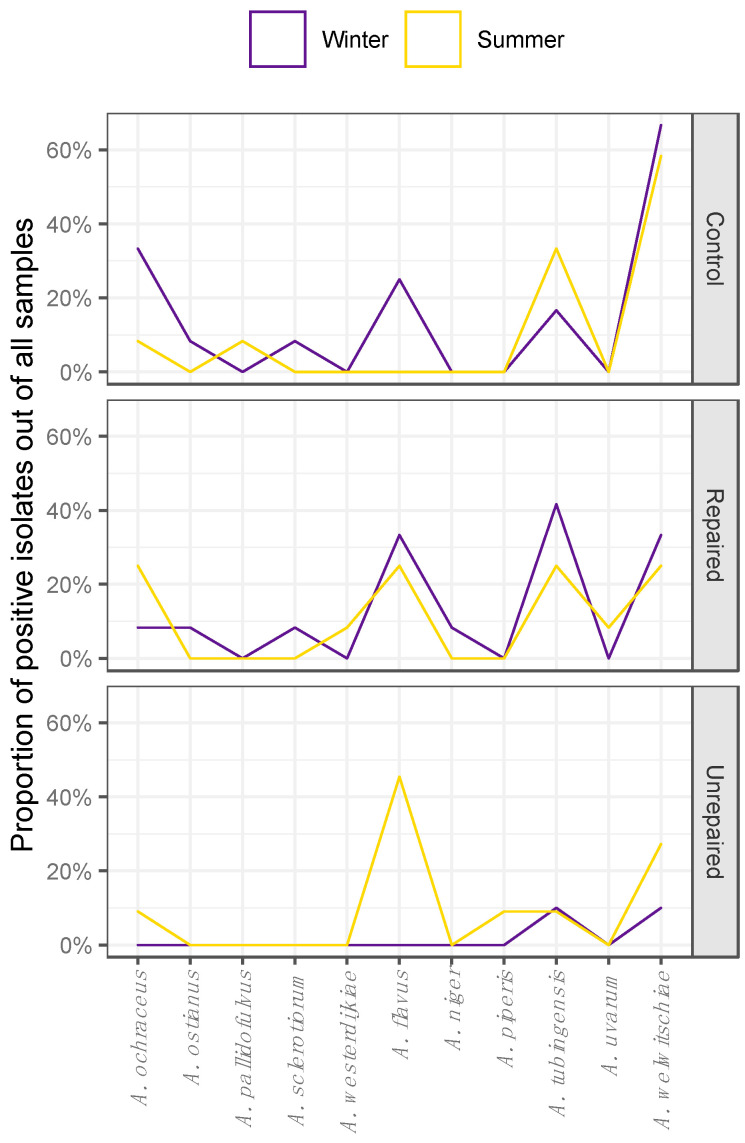
Seasonal distribution patterns of Aspergilli from the sections Circumdati (A. ochraceus, A. ostianus, A. pallidofulvus, A. sclerotiorum, and A. westerdijkiae), Flavi (A. flavus) and Nigri (A. niger, A. piperis, A. tubingensis, A. uvarum, and A. welwitschiae) in dust collected during winter and summer sampling periods in repaired houses, unrepaired houses, and control houses.

The airborne and dust borne Aspergilli followed the different seasonal patterns of species distribution at the post-flood and the control locations as shown on the corresponding area plot diagrams (Figure 3 and Figure 4, respectively). 

The least diverse were the airborne isolates collected at the control locations in the winter sampling period, all of which belonged to the section *Nigri* (Figure 3). At the same location in the summer, airborne Aspergilli were represented by species from all three sections, predominantly by black Aspergilli, which comprised 15.5% of all airborne Aspergilli. While *A. welwitschiae* and *A. tubingensis* were detected in both seasons, *A. piperis* was identified only in winter and *A. niger* was identified only in summer (Figure 3). The airborne Aspergilli from the section *Circumdati* were represented by *A. ochraceus*, *A. ostianus,* and *A. westerdijkiae,* comprising 5.6% of all airborne Aspergilli (Figure 3).

The airborne Aspergilli were the most diverse at the repaired locations, and different seasonal patterns were observed compared to the control locations (Figure 3). Black Aspergilli dominated at this location, while winter isolates of *A. tubingensis* and *A. welwitschiae* comprised 5.6% and 8.5% of airborne Aspergilli. Aspergilli from the section *Circumdati* comprised 7% of airborne Aspergilli and included the species *A. sclerotiorum,* which was not detected at other locations. The proportion of isolates assigned to *A. flavus* (section *Flavi*) remained unchanged from winter to summer at this location (Figure 3), while the species compositions in other sections changed. Although less abundant, black Aspergilli were more diverse in the summer compared to the winter, and there were no isolates of *A. tubingensis*. The section *Circumdati* was less diverse in the summer compared to the winter, and *A. westerdijkiae* was the only species detected.

Airborne isolates from unrepaired locations were more diverse in winter. Most of the isolates belonged to *A. flavus,* which is a particularly dominant species at this location, comprising 12.7% of all airborne Aspergilli. The section *Nigri* was represented by four different species: *A. tubingensis*, *A. welwitschiae, A. niger*, and *A. piperis* (Figure 3). While they were not detected in the winter sampling period, in the summer, the section *Circumdati* comprised 5.6% of airborne Aspergilli, all belonging to *A. ostianus*. The proportion of isolates assigned to *A. flavus* declined compared to winter, while among black Aspergilli, only *A. tubingensis* was detected (Figure 3).

The Aspergilli isolated from dust collected at the control locations in winter were highly diverse, and the isolates from the sections *Circumdati*, *Flavi,* and *Nigri* comprised 7.9%, 4%, and 13%, respectively, of all dust borne Aspergilli. In the summer, the species diversity followed the decline in proportion of dust borne Aspergilli at this location. The only species detected in the section *Circumdati* were *A. ochraceus* and *A. pallidofulvus,* and there were no isolates of *A. flavus*. However, there were more isolates of *A. tubingensis* in the summer than in the winter at this location. 

The Aspergilli in dust collected at repaired locations were more diverse compared to control locations, and different seasonal patterns in species distribution were observed compared to control locations (Figure 4). There were about two times less Aspergilli from the section *Circumdati* in winter compared to the control locations, and there was no difference in the species composition. While *A. ochraceus* was present at both locations in both sampling periods, *A. ostianus* and *A. sclerotiorum* were detected only in the winter. In the summer, *A. pallidofulvus* was detected at the control location and *A. westerdijkiae* at the repaired post-flood location. Isolates assigned to *A. flavus* were detected at both locations but prevailed at the repaired post-flood location, namely in winter, and comprised 5.3% of the total dust borne Aspergilli. *A. welwitschiae* dominated among all dust borne Aspergilli (35%) being 2.5 times more frequent than *A. tubingensis* at control locations. At repaired locations, *A. tubingensis* was slightly more frequent than *A. welwitschiae,* comprising 10.5% and 9.21%, respectively, of total dust borne Aspergilli collected at repaired post-flood locations. In addition, black Aspergilli were more diverse at repaired post-flood locations compared to the control, as *A. niger* and *A. uvarum* were detected at these locations in winter and summer, respectively.

In the dust collected in the winter, only isolates from the section *Nigri* were detected at the unrepaired locations and belonged to *A. tubingensis* and *A. welwitschiae*. Although the dust borne Aspergilli from the section *Flavi* were detected in the samples from unrepaired locations in the winter sampling period (Figure 2; Supplementary Materials, Table S8), they could not be isolated due to overgrowth with *Trichoderma* spp. In the summer, the dust borne Aspergilli were more diverse at this location, namely section *Nigri* (*A. piperis*, *A. tubingensis,* and *A. welwitschie*), while most of the dust borne isolates (6.6%) belonged to *A. flavus.* In addition, the section *Circumdati* was represented by *A. ochraceus* (Figure 4).

## 4. Discussion

The purpose of this study was to explore the occurrence and species distribution of airborne and dust borne Aspergilli from the sections *Circumdati*, *Flavi*, and *Nigri* at post-flood locations, repaired and unrepaired, compared to control locations. In addition, the role of seasonality on species distribution was inspected at each of the sampling locations. This is the first research of its kind conducted in Croatia, and Europe, regarding the presence of these medically and economically important groups of Aspergilli.

While assessing and evaluating fungal contamination in an indoor environment, it is important to take a sample where contamination is suspected along with another sample from a similar but unsuspected location for comparison [48]. Equally important is the sampling method and the sample itself, i.e., air and dust. Among the many available methods of collecting airborne fungi [49], direct impact on nutrient agar is by far the most widely used procedure to assess the concentration and composition of fungi in an indoor environment [50]. Dust samples may be an even more representative indoor sample in the assessment of human exposure to indoor fungi, as they contain indoor fungi, as well as those brought indoors from various outdoor sources [51]. In the presented research, houses and schools were categorised into the same group, i.e., post-flood repaired locations and control locations, as they represent similar indoor environments in regard to the expected fungal contamination [52].

Since water is a major promoter of fungal proliferation [6], the expected aftermath of flooding was more indoor fungi in the affected environment. The concentrations of airborne and dust borne Aspergilli at the post-flood locations were up to twenty times higher compared to the control locations. This observation is in agreement with the previously documented proliferation of Aspergilli after flooding [1,2,3,4,5]. Generally, airborne and dust borne Aspergilli dominate in post-flood indoor environments in the winter, while their concentrations in outdoor air increase in the summer. Decreased concentrations of dust borne fungi during summer months are already known in the literature, and they are explained by an increased ventilation rate [50]. Aspergilli grow better in darkness, and these conditions also support mycotoxin production [53], so an additional explanation may be found in shorter daylight periods and fewer sunny days in the winter compared to the summer. However, in this study, it has been observed that Aspergilli at the unrepaired post-flood locations followed the opposite seasonal pattern from that observed at the repaired or control locations. These houses are vacant, and as such, the air ventilation remains the same throughout the year. In addition, there is no heating, air-conditioning, activities such as cooking and washing, or any other human influence. Thus, this may account for observed differences in seasonal pattern, as these variables contribute to differences in the established equilibrium between indoor and outdoor environments.

The adverse health effects due to exposure to indoor airborne fungi are not fully clarified, but it is generally accepted that fungal rhinitis, hypersensitivity pneumonia, and/or asthma are more prevalent among occupants of water damaged, mouldy environments [54]. For instance, a study conducted following the hurricanes Irene and Sandy on the east coast of the USA demonstrated that the post-hurricane population had become more sensitised and reactive to moulds [55]. Specifically, the portion of Aspergillus-sensitised subjects increased from 15% to 45% in the post-hurricane population [55].

Aspergilli are ubiquitous fungi, and their presence in indoor and outdoor environments is not abnormal per se. Presented results have shown elevated concentrations of Aspergilli at control locations in certain sampling periods, e.g., dust borne *Circumdati* in the winter sampling period, or airborne *Nigri* in the summer period of sampling, while Aspergilli from the section *Flavi* always prevailed at post-flood locations. 

In the case of the section *Circumdati,* the important observation was that their concentrations dropped in the summer, and that they were not detected in indoor air at the same location in the same season. Although *A. ochraceus* was most frequently identified among the *Circumdati* isolates, and its capacities to produce OTA were previously reported [56], the only OTA-producing species was *A. westerdijkiae*, which is most frequently isolated at post-flood locations. The amount of OTA detected in corresponding fungal extracts was 13.7 ± 15.81 µg/mL (Table 1, Supplementary Materials, Table S7). The isolates of *A. sclerotiorum* detected in air and dust samples of post-flood locations and control locations did not produce OTA, while in a previous study conducted in Croatia, the concentrations of OTA detected in spore extracts of *A. sclerotiorum* were 0.3–28 µg/mL [57]. Other species detected among the airborne and dust borne isolates included *A. ostianus* and *A. pallidofulvus,* which did not produce OTA, although according to the literature, some isolates of *A. ostianus* may produce OTA in trace amounts [56]. To the best of our knowledge, this is the first report of *A. pallidofulvus* in the indoor environment. It was described as a separate species from *A. ochraceus* in 2014, and its origins were green coffee beans from India as well as two unrecorded sources [56]. More recently, it was isolated from Pu-erh tea [58]. Previously, *A. westerdijkiae* was reported in high amounts in house dust from South Africa and air samples from The Netherlands [56], and *A. ochraceus* was reported in floor-related material and the wet-concrete of water-damaged buildings [11], while *A. ostianus* was detected in indoor air in Denmark [56]. Severe health hazards linked to the inhalation of OTA have been described in occupational settings, i.e., in a granary where workers exposed to OTA-producing Aspergilli suffered from acute renal failure [59]. Although studies about the exposure to mycotoxins in indoor environment are rare, OTA was detected in the urine of occupants in water-damaged buildings and associated with focal segmental glomerulosclerosis [60]. Furthermore, all of the identified species may be associated with infections, including skin and nail infections [61] but also allergic bronchopulmonary aspergillosis and otomycosis [56].

In the presented study, *A. flavus* was identified almost exclusively at the post-flood locations, where it comprised 17–25% of total airborne Aspergilli in the winter and 7–13% in the summer (Figure 1, Figure 3). All isolates assigned to the section *Flavi* were identified as *A. flavus,* and 7/33 isolates produced AFB_1_ in concentrations of 2.51 ± 5.31 µg/mL (Table 1, Supplementary Materials, Table S8). None of the AFB_1_-producing *A. flavus* were detected at the control locations. Interestingly, a single AFB_1_-producing isolate from a repaired post-flood location was recovered from an outdoor air sample. In previous research on indoor Aspergilli in living and occupational environments in Croatia, *A. parasiticus* was the only other species, besides *A. flavus,* detected among airborne isolates [19]. In other studies, some of the isolates from house dust were assigned to other species from this section, including *A. tamarii*, *A. nomius,* and *A. pseudonomius* [16]. The dominant association of aflatoxigenic *A. flavus* with water-damaged environments highlights a need for immediate action, i.e., the remediation of unrepaired locations, as they may serve as a sink for their proliferation and spread through the surrounding area.

Exposure to AFB_1_ via inhalation was investigated in industrial settings, where it was associated with pulmonary interstitial fibrosis in agricultural and textile workers [62]. Its role in primary liver cancer was supported by detectable levels of AFB_1_ in the serum of about 60% of the exposed workers in poultry farms and none of the control subjects [63]. This species is the second leading cause of invasive aspergillosis and is associated with approximately 10% of bronchopulmonary aspergillosis [64]. It may cause various diseases, including keratomycosis [65] and endophthalmitis [66].

Interesting results were obtained for the Aspergilli from the section *Nigri*. They were the most frequent among the three sections of interest and prevailed at post-flood locations, where they comprised 33% of airborne and 28% of dust borne Aspergilli. Most of the isolates were identified as *A. tubingensis* and *A. welwitschiae,* in agreement with previously conducted research on black Aspergilli in indoor air [17,18]. Mycotoxin FB_2_ was produced by *A. niger* and *A. welwitschiae,* while the concentrations of FB_2_ detected in corresponding fungal extracts were 11.24 ± 18.30 µg/mL and 6.76 ±13.51 µg/mL, respectively (Table 1, Supplementary Materials, Table S9). Despite a relatively small sample, our results are in agreement with the conclusions of previous studies, according to which a majority of *A. niger* and a minority of *A. welwitschiae* isolates produce FB_2_ [67]. The concentrations of black Aspergilli in indoor air samples at control locations were several times higher than those measured at the post-flood locations, and they were also elevated compared to outdoor air samples. This observation suggests an inner source of contamination with black Aspergilli at some of the control locations. The most common species among airborne and dust borne black Aspergilli is *A. welwitschiae,* and at the control locations, it prevailed over *A. tubingensis,* which was much more frequent at the post-flood locations. It was shown that a decline in water activities negatively affects the growth of *A. tubingensis* [68], and it may explain why this species prevailed at the post-flood locations over control locations, especially in the winter sampling period. The inability of *A. tubingensis* to produce mycotoxins could have implied a lower risk for the occupants compared to mycotoxin-producing *A. welwitschiae*. However, *A. tubingensis* is capable of producing other biologically active metabolites, including naphto-y-pyrones asperazine and malformins [69], which may contribute to deleterious effects in affected tissue. Furthermore, some studies in the clinical settings, where both *A. welwitschiae* and *A. tubingensis* are frequently isolated, [70,71,72] have shown that *A. tubingensis* is associated with the chronic pulmonary aspergillosis, while *A. welwitschiae* is suspected to be more related to fungal colonisation than infection [73]. Regarding other species from the section *Nigri,* this is the first report of *A. uvarum* in an indoor environment in Croatia. It was isolated from indoor air and dust samples from post-flood repaired locations in the summer sampling season. In 2008, it was introduced in the section *Nigri* and isolated from grape berries from multiple Southern European countries [74]. Later, it was reported in indoor and outdoor air samples in the United States [17], as well as indoor air samples from Turkey [18]. In this study, *A. niger* was isolated from indoor and outdoor air samples at post-flood locations in the winter and control locations in the summer, in addition to a single dust borne isolate collected at a repaired location in the winter. Airborne and dust borne isolates of *A. piperis* were collected at post-flood and control locations in both seasons. Both *A. niger* and *A. piperis* were previously reported as indoor air isolates from Croatia, while *A. niger* was isolated from indoor air in Hungary, The Netherlands, and Thailand [18]. 

## 5. Conclusions

The results of the presented study indicate that houses and schools, especially after water damage, may be important sources of various Aspergilli that are recognised human pathogens. In addition, this research provides valuable information regarding the composition of important mycotoxin-producing and non-producing Aspergilli at water-damaged locations and control locations, which is a study that is the first of its kind in Croatia and Europe. The exclusive presence of aflatoxigenic species *A. flavus* at the post-flood locations is the most worrying finding and infers the need for a thorough remediation. New genotyping strategies that would link environmental and clinical isolates could be of great importance for a better understanding of the significance of *Aspergillus* exposure after flooding events. The role of mycotoxins in adverse health effects upon inhalation remains a subject of debate. More information regarding their detection in clinically relevant samples supported by their presence in living and occupational settings might bring us closer to understanding their role in disease.

## Figures and Tables

**Table 1:** Mycotoxin-producing abilities of the species assigned to the sections *Circumdati, Flavi,* and *Nigri,* and the distribution of mycotoxin-producing isolates over control and post-flood locations.

Species	Mycotoxin	*n*/*N*	Concentrations of a Mycotoxin in Fungal Extract (µg/mL)	CL—RL—UNRL	
			Mean ± SD	Air-Samples	Dust
*A. westerdijkiae*	OTA	4/5	13.7 ± 15.81	1—2—0	0—1—0
*A. flavus*	AFB_1_	7/33	2.51 ± 5.31	0—1—3	0—0—3
*A. niger*	FB_2_	3/5	11.24 ± 18.30	2—0—0	0—1—0
*A. welwitschiae*	FB_2_	10/41	6.76 ± 13.51	0—2—0	6—1—1

*n*/*N*—number of mycotoxin-producing isolates/number of total isolates; CL—RL—UNRL—breakdown number of mycotoxin-producing isolates detected at each location in air-samples and dust; CL—control locations; RL—repaired post-flood locations; UNRL—unrepaired post-flood locations;.

## Supplementary Materials

The following are available online at https://www.mdpi.com/2309-608X/6/4/282/s1, Tables S1–S3: Concentrations of airborne Aspergilli from the section *Circumdati* (Table S1), *Flavi* (Table S2) and *Nigri* (Table S3) (CFU/m^3^) isolated from indoor air samples during winter and summer sampling periods at the locations specified; Tables S4–S6: Concentrations of Aspergilli from the section *Circumdati* (Table S4), *Flavi* (Table S5) and *Nigri* (Table S6) in dust (CFU/g) collected during winter and summer sampling periods at the locations specified. Table S7: Ochratoxin A (OTA)-producing abilities of airborne and dust-borne Aspergilli from the section *Circumdati;* Table S8. Aflatoxin B_1_ (AFB_1_)-producing abilities of airborne and dust-borne Aspergilli from the section *Flavi*: Table S9: Fumonisin B_2_ (FB_2_)-producing abilities of airborne and dust-borne Aspergilli from the section *Nigri*; Figure S1–S3: Phylogenetic trees based on the datasets of *CaM* sequences showing the relationship between isolates assigned to *Aspergillus* section *Circumdati* (Figure S1), *Flavi* (Figure S2), and *Nigri* (Figure S3). The bootstrap percentages of the maximum likelihood (ML) analysis are presented at the nodes; The bar indicates the number of substitutions per site. The phylogram is rooted with *Aspergillus tanneri* NRRL 62425 (Figure S1), *Aspergillus flavipes* NRRL 302^T^ (Figure S2), and *A. nidulans* NRRL 187^T^ (Figure S3). Figure S4: HPLC-ESI-MS total ion current chromatogram of selected extract of an OTA-producing isolate of *A. westerdijkiae* MFBF AC12375, RT (OTA) = 8.8 min. (**a**); corresponding HPLC-ESI-MS spectrum (**b**) and MS2 spectra (**c**); Concentration of OTA in the extract was 31.50 µg/mL and extrapolated from OTA standard calibration line constructed by plotting the peak area of *m/z* 260.9 obtained from HPLC-ESI-MS/MS extracted ion chromatogram vs. OTA concentration; HPLC-ESI-MS spectrum of OTA standard (**d**) and HPLC-ESI-MS/MS fragmentation pattern of a major ion *m*/*z* 426.2 [M+Na]^+^(**e**). Figure S5: HPLC-ESI-MS total ion current chromatogram of selected extract of AFB_1_-producing *A. flavus* isolate MFBF AF12404 (**a**); corresponding HPLC-ESI-MS extracted ion chromatogram (EIC) of major ions *m*/*z* 313.0 [MH]^+^ (black, higher peak) and *m*/*z* 335.0 [MNa]^+^ (light gray, shorter peak) at RT 5.2 min. Concentration of AFB_1_ in the extract was 14.52 µg/mL and extrapolated from AFB_1_ standard calibration line constructed by plotting the peak area of *m/z* 313 obtained from extracted ion chromatogram vs. AFB_1_ concentration (**b**); HPLC-ESI-MS spectrum of AFB_1_ standard (**c**). Figure S6: HPLC-ESI-MS total ion current chromatogram of selected extract of a FB_2_-producing isolate of *A. niger* MFBF AN12137 (light gray) overlaid with extracted ion m/z 706 chromatogram at RT 8.4 min (black) (**a**) and corresponding MS2 spectra (**b**). Concentration of FB_2_ in the extract was 32.383 µg/mL and extrapolated from FB2 standard calibration line constructed by plotting the peak area of m/z 336.2 obtained from HPLC-ESI-MS/MS extracted ion chromatogram vs FB_2_ concentration; HPLC-ESI-MS spectrum of FB2 standard (**c**) and HPLC-ESI-MS/MS fragmentation pattern of a major molecular ion [M+H]+ m/z 706 (**d**).

## Appendix A

### Parameters for LC/MS Analysis

Prior to use, the mobile phases were filtered through a cellulose nitrate filter, diameter 47 mm, pore size 0.45 µm (Sartorius, Goettingen, Germany). Degassing of the mobile phase was carried out continuously with an on-line degasser. The samples and the standards were kept in glass (OTA, AFB_1_) and polypropylene (FB_2_) HPLC vials at 4 °C in the autosampler temperature control system, and in each analysis, 10 µL were injected. Symmetry C18, 4.6 × 150 mm (3.5 µm) column (Agilent Technologies, Waldbronn, Germany) was used as the stationary phase in all performed chromatographic separations, incubated at 35.0 ± 0.1 °C (OTA and FB_2_) and 40.0 ± 0.1 °C (AFB_1_). A separation of OTA was performed in isocratic mode at flow rate of 0.6 mL/min, while the mobile phase consisted of MeOH:H_2_O:AcOH = 70:30:0.1% (*v/v*). Separation of AFB_1_ and FB_2_ followed the gradient protocol. The mobile phase set at the flow rate of 0.5 mL/min (for AFB_1_) and 0.7 mL/min (FB_2_) consisted of MeCN: H_2_O:HCOOH = 50:50:0.1% (*v/v*) as eluent A (for AFB_1_ and FB_2_), while eluent B was MeCN for AFB_1_ and H_2_O:HCOOH = 100:0.1% (*v/v*) for FB_2_. The time program to elute AFB_1_ was 100% A (0–6 min), 100→90% A (6–8 min), 90% A (8–10 min), 90→100% A (10–12 min), and 100% A (12–14 min). In the analysis of FB_2_, the time program was 75% B (0–1 min), 75 → 60% B (1–5 min), 60% B (5–10 min), 75 → 60% B (10–12 min), and 75% B (12–17 min).

Mass spectrometry analysis were carried out with electrospray ionisation (ESI) source in the positive ion mode. The entire effluent was delivered to the mass spectrometer. Nitrogen was used both as drying gas at a flow rate of 6.0 L/min (OTA, AFB_1_), 10.0 L/min (FB_2_), and as nebulizer gas at a pressure of 10.0 psi (OTA, AFB_1_) and 15.0 psi (FB_2_). To maximize method sensitivity, the electrospray source temperature was optimized at 325 °C, and the capillary voltage was set at 3.5 kV. The full scan mass spectra were acquired over a range *m/z* 100–800 with max accumulation time of 200 ms. Quantitative analysis was performed in Selected reaction monitoring (SRM) mode by monitoring the transition *m/z* 426.0 → 260.9 (OTA), *m/z* 313 (AFB_1_) and 706.0 → 336.0 (FB_2_).
