# Supplementary material for: Post-Flood Impacts on Occurrence and Distribution of Mycotoxin-Producing Aspergilli from the Sections Circumdati, Flavi, and Nigri in Indoor Environment
Source: J Fungi (Basel). 2020 Nov 12;6(4):282. doi: 10.3390/jof6040282 (PMC7711759; doi:10.3390/jof6040282)
Supplement: Supplementary file 1 [file jof-06-00282-s001.zip › Supplementary Materials/Figures S4S5S6.docx]

| **a** |
| --- |
| **b** |
| **c** |
| d |
| e |
| **Figure S4.** HPLC-ESI-MS total ion current chromatogram of selected extract of an OTA-producing isolate of *A. westerdijkiae* MFBF AC12375, RT (OTA) = 8.8 min. (**a**); corresponding HPLC-ESI-MS spectrum (**b**) and MS2 spectra (**c**); Concentration of OTA in the extract was 31.50 µg/ml and extrapolated from OTA standard calibration line constructed by plotting the peak area of *m/z* 260.9 obtained from HPLC-ESI-MS/MS extracted ion chromatogram *vs* OTA concentration; HPLC-ESI-MS spectrum of OTA standard (**d**) and HPLC-ESI-MS/MS fragmentation pattern of a major ion *m*/*z* 426.2 [M+Na]^+^(**e**). |

| a |
| --- |
| b |
| c |
| **Figure S5.** HPLC-ESI-MS total ion current chromatogram of selected extract of AFB_1_ producing *A. flavus* isolate MFBF AF12404 (**a**); corresponding HPLC-ESI-MS extracted ion chromatogram (EIC) of major ions *m*/*z* 313.0 [MH]^+^(black, higher peak) and *m*/*z* 335.0 [MNa]^+^ (light gray, shorter peak) at RT 5.2 min. Concentration of AFB_1_ in the extract was 14.52 µg/ml and extrapolated from AFB_1_ standard calibration line constructed by plotting the peak area of *m/z* 313 obtained from extracted ion chromatogram vs. AFB_1_ concentration (**b**); HPLC-ESI-MS spectrum of AFB_1_ standard (**c**). |

| a |
| --- |
| b  |
| c |
| d |
| **Figure S6.** HPLC-ESI-MS total ion current chromatogram of selected extract of a FB_2_-producing isolate of *A. niger* MFBF AN12137 (light gray) overlaid with extracted ion *m/z* 706 chromatogram at RT 8.4 min (black) (**a**) and corresponding MS2 spectra (**b**). Concentration of FB_2_ in the extract was 32.383 µg/ml and extrapolated from FB_2_ standard calibration line constructed by plotting the peak area of *m/z* 336.2 obtained from HPLC-ESI-MS/MS extracted ion chromatogram *vs* FB_2_ concentration; HPLC-ESI-MS spectrum of FB_2_ standard (**c**) and HPLC-ESI-MS/MS fragmentation pattern of a major molecular ion [M+H]^+^ *m/z* 706 (**d**). |
