# Supplementary material for: Post-Flood Impacts on Occurrence and Distribution of Mycotoxin-Producing Aspergilli from the Sections Circumdati, Flavi, and Nigri in Indoor Environment
Source: J Fungi (Basel). 2020 Nov 12;6(4):282. doi: 10.3390/jof6040282 (PMC7711759; doi:10.3390/jof6040282)
Supplement: Supplementary file 1 [file jof-06-00282-s001.zip › Supplementary Materials/Table S1toS9_final.docx]

**Table S1.** Concentrations of airborne Aspergilli from the section *Circumdati* (CFU/m^3^) isolated from indoor air samples during winter and summer sampling period at the locations specified.

|  | **Winter** | | | **Summer** | | |
| --- | --- | --- | --- | --- | --- | --- |
|  | Unrepaired locations | Repaired locations | Control locations | Unrepaired locations * | Repaired locations | Control locations |
| n/N | 0/40 | 5/60 | 0/60 | 9/40 | 2/40 | 5/40 |
|  | CFU/m^3^ | | | | | |
| Min | - | 20 | - | 20 | 20 | 20 |
| Max | - | 20 | - | 60 | 20 | 40 |
| M | - | 20 | - | 40 | - | 20 |

n/N – number of positive samples / total number of samples; Min- minimum CFU/m^3^ detected, Max- maximum CFU/m^3^ detected; M- median CFU/m^3^ detected; * statistically significant difference compared to the Repaired locations within the same season (p < 0.001).

**Table S2.** Concentrations of airborne Aspergilli from the section *Flavi* (CFU/m^3^) isolated from indoor and outdoor air samples during winter and indoor air in summer sampling period at the locations specified.

|  | **Winter** | | | | | | **Summer** | | |
| --- | --- | --- | --- | --- | --- | --- | --- | --- | --- |
|  | Indoor | | | Outdoor | | | Indoor | | |
|  | Unrepaired locations * | Repaired locations | Control locations | Unrepaired locations | Repaired locations | Control locations | Unrepaired locations | Repaired locations | Control locations |
| n/N | 8/40 | 1/60 | 0/60 | 1/10 | 2/12 | 0/12 | 4/40 | 3/60 | 1/60 |
|  | CFU/m^3^ | | | | | | | | |
| Min | 20 | - | - | 20 | 20 | - | 20 | 20 | 40 |
| Max | 40 | - | - | - | 20 | - | 40 | 80 | - |
| M | 20 | 20 | - | - | - | - | 20 | 20 | - |

n/N – number of positive samples / total number of samples; Min- minimum CFU/m^3^ detected, Max- maximum CFU/m^3^ detected; M- median CFU/m^3^ detected; * - statistically significant difference compared to the Repaired locations within the same season (p = 0.001)

**Table S3.** Concentrations of airborne Aspergilli from the section *Nigri* (CFU/m^3^) isolated from indoor and outdoor air samples during winter and summer sampling period at the locations specified.

|  | **Winter** | | | | | | **Summer** | | | | | |
| --- | --- | --- | --- | --- | --- | --- | --- | --- | --- | --- | --- | --- |
|  | Indoor | | | Outdoor | | | Indoor | | | Outdoor | | |
|  | Unrepaired locations | Repaired locations | Control locations | Unrepaired locations | Repaired locations | Control locations | Unrepaired locations | Repaired locations | Control locations | Unrepaired locations | Repaired locations | Control locations |
| n/N | 5/40 | 9/60 | 4/60 | 2/10 | 1/12 | 0/12 | 3/40 | 2/60 | 11/60 | 1/10 | 0/12 | 2/12 |
|  | CFU/m^3^ | | | | | | | | | | | |
| Min | 20 | 20 | 20 | 40 | 20 | 0 | 20 | 20 | 20 | 100 | - | 20 |
| Max | 60 | 1100 | 40 | 1980 | - | 0 | 60 | 80 | 680 | - | - | 20 |
| M | 20 | 40 | 30 | - | - | - | 60 | -- | 40 | - | - | - |

n/N – number of positive samples / total number of samples; Min- minimum CFU/m^3^ detected, Max- maximum CFU/m^3^ detected; M- median CFU/m^3^ detected.

**Table S4.** Concentrations of Aspergilli from the section *Circumdati* in dust (CFU/g) collected during winter and summer sampling period at the locations specified.

|  | **Winter** | | | **Summer** | | |
| --- | --- | --- | --- | --- | --- | --- |
|  | Control locations | Repaired locations | Unrepaired locations | Control locations | Repaired locations | Unrepaired locations |
| n/N | 6/12 | 4/12 | 1/10 | 2/12 | 5/12 | 1/10 |
|  | CFU/g | | | | | |
| Min | 82 | 83 | 600 | 45 | 45 | 893 |
| Max | 42727 | 600 | - | 50 | 619 | - |
| M | 1745 | 300 | - | - | 100 | - |

n/N – number of positive samples / total number of samples; Min- minimum CFU/g detected; % - proportion from total number of the dust-borne Aspergilli detected at each location; of Max- maximum CFU/g detected; M- median CFU/g detected

**Table S5.** Concentrations of Aspergilli from the section *Flavi* in dust (CFU/g) collected during winter and summer sampling period at the locations specified.

|  | **Winter** | | | **Summer** | | |
| --- | --- | --- | --- | --- | --- | --- |
|  | Control locations | Repaired locations | Unrepaired locations | Control locations | Repaired locations | Unrepaired locations |
| n/N | 3/12 | 2/12 | 1/10 | 0/12 | 3/12 | 6/10 |
|  | CFU/g | | | | | |
| Min | 90 | 200 | 200 | 0 | 200 | 45 |
| Max | 150 | 545 | - | 0 | 500 | 17568 |
| M | 100 | - | - |  | 364 | 146 |

n/N – number of positive samples / total number of samples; Min- minimum CFU/g detected; Max- maximum CFU/g detected; M- median CFU/g detected.

**Table S6.** Concentrations of Aspergilli from the section *Nigri* in dust (CFU/g) collected during winter and summer sampling period at the locations specified.

|  | **Winter** | | | **Summer** | | |
| --- | --- | --- | --- | --- | --- | --- |
|  | Control locations | Repaired locations | Unrepaired locations | Control locations | Repaired locations | Unrepaired locations |
| n/N | 8/12 | 6/12 | 2/10 | 8/12 | 7/12 | 5/10 |
|  | CFU/g | | | | | |
| Min | 90 | 45 | 100 | 45 | 45 | 45 |
| Max | 4545 | 1818 | 200 | 364 | 1773 | 571 |
| M | 1024 | 696 | - | 100 | 524 | 143 |

n/N – number of positive samples / total number of samples; Min- minimum CFU/g detected Max- maximum CFU/g detected; Mean ± SD – average CFU/g detected with standard deviation; M- median CFU/g detected

**Table S7.** Ochratoxin A (OTA) producing abilities of airborne and dust-borne Aspergilli from the section *Circumdati.*

| **Species** | **Isolate code** | **Sampling period** | **Source** | | **Location** | **OTA µg/ml** |
| --- | --- | --- | --- | --- | --- | --- |
| *A. ochraceus* | MFBF AC12382 | Summer | indoor air | | Control locations |  |
| *A. ochraceus* | MFBF AC12701 | Summer | indoor air | | Control locations |  |
| *A. ochraceus* | MFBF AC12122 | Winter | indoor air | | Repaired locations |  |
| *A. ostianus* | MFBF AC12706 | Summer | indoor air | | Control locations |  |
| *A. ostianus* | MFBF AC12651 | Summer | indoor air | | Unrepaired locations |  |
| *A. ostianus* | MFBF AC12652 | Summer | indoor air | | Unrepaired locations |  |
| *A. ostianus* | MFBF AC12653 | Summer | indoor air | | Unrepaired locations |  |
| *A. ostianus* | MFBF AC12654 | Summer | indoor air | | Unrepaired locations |  |
| *A. sclerotiorum* | MFBF AC12077 | Winter | indoor air | | Repaired locations |  |
| *A. sclerotiorum* | MFBF AC12079 | Winter | indoor air | | Repaired locations |  |
| *A. westerdijkiae* | MFBF AC12375 | Summer | indoor air | | Control locations | 31.5 |
| *A. westerdijkiae* | MFBF AC12124 | Winter | indoor air | | Repaired locations | 0.46 |
| *A. westerdijkiae* | MFBF AC12125 | Winter | indoor air | | Repaired locations | 0.29 |
| *A. westerdijkiae* | MFBF AC12737 | Summer | indoor air | | Repaired locations |  |
|  |  | N producing airborne isolates/N total airborne isolates | | | | 3/14 |
| *A. ochraceus* | MFBF AC12210 | Winter | dust | | Control locations |  |
| *A. ochraceus* | MFBF AC12413 | Summer | dust | | Control locations |  |
| *A. ochraceus* | MFBF AC12564 | Winter | dust | | Control locations |  |
| *A. ochraceus* | MFBF AC12566 | Winter | dust | | Control locations |  |
| *A. ochraceus* | MFBF AC12568 | Winter | dust | | Control locations |  |
| *A. ochraceus* | MFBF AC12143 | Winter | dust | | Repaired locations |  |
| *A. ochraceus* | MFBF AC12685 | Summer | dust | | Repaired locations |  |
| *A. ochraceus* | MFBF AC12688 | Summer | dust | | Repaired locations |  |
| *A. ochraceus* | MFBF AC12689 | Summer | dust | | Repaired locations |  |
| *A. ochraceus* | MFBF AC12404 | Summer | dust | | Unrepaired locations |  |
| *A. ostianus* | MFBF AC12567 | Winter | dust | | Control locations |  |
| *A. ostianus* | MFBF AC12138 | Winter | dust | | Repaired locations |  |
| *A. pallidofulvus* | MFBF AC12744 | Summer | dust | | Control locations |  |
| *A. sclerotiorum* | MFBF AC12208 | Winter | dust | | Control locations |  |
| *A. sclerotiorum* | MFBF AC12510A | Winter | dust | | Repaired locations |  |
| *A. westerdijkiae* | MFBF AC12408 | Summer | dust | | Repaired locations | 22.55 |
|  |  | N producing dust-borne isolates/N total dust-borne isolates | | | | 1/16 |
|  |  |  | | | |  |
| OTA-producing species |  | N producing isolates/N total isolates | | Concentration (µg/ml) arithmetic mean ± SD | | |
| *A. westerdijkiae* |  | 4/5 | | 13.7 ± 15.81 | | |

**Table S8.** Aflatoxin B_1_ (AFB_1_) producing abilities of airborne and dust-borne Aspergilli from the section *Flavi.*

| **Species** | **Isolate Code** | **Sampling Period** | **Source** | | **Location** | **AFB_1_ µg/ml** |
| --- | --- | --- | --- | --- | --- | --- |
| *A. flavus* | MFBF AF12119 | Winter | indoor air | | Repaired locations |  |
| *A. flavus* | MFBF AF12640 | Summer | indoor air | | Repaired locations |  |
| *A. flavus* | MFBF AF12676 | Summer | indoor air | | Repaired locations |  |
| *A. flavus* | MFBF AF12088 | Winter | indoor air | | Unrepaired locations |  |
| *A. flavus* | MFBF AF12431 | Winter | indoor air | | Unrepaired locations |  |
| *A. flavus* | MFBF AF12432 | Winter | indoor air | | Unrepaired locations |  |
| *A. flavus* | MFBF AF12435 | Winter | indoor air | | Unrepaired locations |  |
| *A. flavus* | MFBF AF12436 | Winter | indoor air | | Unrepaired locations | 0.25 |
| *A. flavus* | MFBF AF12441 | Winter | indoor air | | Unrepaired locations |  |
| *A. flavus* | MFBF AF12443 | Winter | indoor air | | Unrepaired locations | 0.05 |
| *A. flavus* | MFBF AF12452 | Winter | indoor air | | Unrepaired locations |  |
| *A. flavus* | MFBF AF12458 | Winter | indoor air | | Unrepaired locations | 0.28 |
| *A. flavus* | MFBF AF12645 | Summer | indoor air | | Unrepaired locations |  |
| *A. flavus* | MFBF AF12648 | Summer | indoor air | | Unrepaired locations |  |
| *A. flavus* | MFBF AF12483 | Winter | outdoor air | | Repaired locations | 1.07 |
| *A. flavus* | MFBF AF12496 | Winter | outdoor air | | Repaired locations |  |
| *A. flavus* | MFBF AF12631 | Summer | outdoor air | | Repaired locations |  |
| *A. flavus* | MFBF AF12660 | Summer | outdoor air | | Unrepaired locations |  |
|  |  | N producing airborne isolates/N total airborne isolates | | | | 4/18 |
| *A. flavus* | MFBF AF12206 | Winter | dust | | Control locations |  |
| *A. flavus* | MFBF AF12565 | Winter | dust | | Control locations |  |
| *A. flavus* | MFBF AF12566 | Winter | dust | | Control locations |  |
| *A. flavus* | MFBF AF12140 | Winter | dust | | Repaired locations |  |
| *A. flavus* | MFBF AF12398 | Summer | dust | | Repaired locations |  |
| *A. flavus* | MFBF AF12510 | Winter | dust | | Repaired locations |  |
| *A. flavus* | MFBF AF12513A | Winter | dust | | Repaired locations |  |
| *A. flavus* | MFBF AF12513B | Winter | dust | | Repaired locations |  |
| *A. flavus* | MFBF AF12685 | Summer | dust | | Repaired locations |  |
| *A. flavus* | MFBF AF12686 | Summer | dust | | Repaired locations |  |
| *A. flavus* | MFBF AF12403B | Summer | dust | | Unrepaired locations | 0.39 |
| *A. flavus* | MFBF AF12404 | Summer | dust | | Unrepaired locations | 14.52 |
| *A. flavus* | MFBF AF12405 | Summer | dust | | Unrepaired locations | 1.07 |
| *A. flavus* | MFBF AF12691 | Summer | dust | | Unrepaired locations |  |
| *A. flavus* | MFBF AF12693 | Summer | dust | | Unrepaired locations |  |
|  |  | N producing dust-borne isolates/N total dust-borne isolates | | | | 3/15 |
|  |  |  | | | |  |
| AFB1 producing species | | N producing isolates/N total isolates | | Concentration (µg/ml) arithmetic mean ± SD | | |
| *A. flavus* | | 7/33 | | 2.51 ± 5.31 | | |

**Table S9.** Fumonisin B_2_ (FB_2_) producing abilities of airborne and dust-borne Aspergilli from the section *Nigri.*

| **Species** | **Isolate Code** | **Sampling Period** | **Source** | **Location** | | **FB_2_ µg/ml** |
| --- | --- | --- | --- | --- | --- | --- |
| *A. niger* | MFBF AN12371 | Summer | indoor air | Control locations | |  |
| *A. niger* | MFBF AN12711 | Summer | indoor air | Control locations | | 1.18 |
| *A. niger* | MFBF AN12436 | Winter | indoor air | Unrepaired locations | |  |
| *A. piperis* | MFBF AN12183 | Winter | indoor air | Control locations | |  |
| *A. piperis* | MFBF AN12614 | Summer | indoor air | Repaired locations | |  |
| *A. piperis* | MFBF AN12443 | Winter | indoor air | Unrepaired locations | |  |
| *A. tubingensis* | MFBF AN12185 | Winter | indoor air | Control locations | |  |
| *A. tubingensis* | MFBF AN12721 | Summer | indoor air | Control locations | |  |
| *A. tubingensis* | MFBF AN12060 | Winter | indoor air | Repaired locations | |  |
| *A. tubingensis* | MFBF AN12493 | Winter | indoor air | Repaired locations | |  |
| *A. tubingensis* | MFBF AN12340 | Summer | indoor air | Unrepaired locations | |  |
| *A. tubingensis* | MFBF AN12448 | Winter | indoor air | Unrepaired locations | |  |
| *A. tubingensis* | MFBF AN12644 | Summer | indoor air | Unrepaired locations | |  |
| *A. tubingensis* | MFBF AN12645 | Summer | indoor air | Unrepaired locations | |  |
| *A. tubingensis* | MFBF AN12646 | Summer | indoor air | Unrepaired locations | |  |
| *A. tubingensis* | MFBF AN12648 | Summer | indoor air | Unrepaired locations | |  |
| *A. uvarum* | MFBF AN12310 | Summer | indoor air | Repaired locations | |  |
| *A. welwitschiae* | MFBF AN12194 | Winter | indoor air | Control locations | |  |
| *A. welwitschiae* | MFBF AN12384 | Summer | indoor air | Control locations | |  |
| *A. welwitschiae* | MFBF AN12706 | Summer | indoor air | Control locations | |  |
| *A. welwitschiae* | MFBF AN12707 | Summer | indoor air | Control locations | |  |
| *A. welwitschiae* | MFBF AN12708 | Summer | indoor air | Control locations | |  |
| *A. welwitschiae* | MFBF AN12709 | Summer | indoor air | Control locations | |  |
| *A. welwitschiae* | MFBF AN12717 | Summer | indoor air | Control locations | |  |
| *A. welwitschiae* | MFBF AN12070 | Winter | indoor air | Repaired locations | |  |
| *A. welwitschiae* | MFBF AN12468 | Winter | indoor air | Repaired locations | | 0.02 |
| *A. welwitschiae* | MFBF AN12473 | Winter | indoor air | Repaired locations | |  |
| *A. welwitschiae* | MFBF AN12474 | Winter | indoor air | Repaired locations | |  |
| *A. welwitschiae* | MFBF AN12475 | Winter | indoor air | Repaired locations | |  |
| *A. welwitschiae* | MFBF AN12476 | Winter | indoor air | Repaired locations | |  |
| *A. welwitschiae* | MFBF AN12626 | Summer | indoor air | Repaired locations | | 1.58 |
| *A. welwitschiae* | MFBF AN12454 | Winter | indoor air | Unrepaired locations | |  |
| *A. niger* | MFBF AN12704 | Summer | outdoor air | Control locations | | 0.18 |
| *A. tubingensis* | MFBF AN12728 | Summer | outdoor air | Control locations | |  |
| *A. tubingensis* | MFBF AN12064 | Winter | outdoor air | Repaired locations | |  |
| *A. tubingensis* | MFBF AN12069 | Winter | outdoor air | Repaired locations | |  |
| *A. tubingensis* | MFBF AN12095 | Winter | outdoor air | Unrepaired locations | |  |
| *A. tubingensis* | MFBF AN12100 | Winter | outdoor air | Unrepaired locations | |  |
| *A. tubingensis* | MFBF AN12649 | Summer | outdoor air | Unrepaired locations | |  |
|  |  |  | N producing airborne isolates/N total airborne isolates | | | 4/39 |
| *A. niger* | MFBF AN12137 | Winter | dust | Repaired locations | | 32.38 |
| *A. piperis* | MFBF AN12404 | Summer | dust | Unrepaired locations | |  |
| *A. tubingensis* | MFBF AN12209 | Winter | dust | Control locations | |  |
| *A. tubingensis* | MFBF AN12410 | Summer | dust | Control locations | |  |
| *A. tubingensis* | MFBF AN12411 | Summer | dust | Control locations | |  |
| *A. tubingensis* | MFBF AN12414 | Summer | dust | Control locations | |  |
| *A. tubingensis* | MFBF AN12563 | Winter | dust | Control locations | |  |
| *A. tubingensis* | MFBF AN12741 | Summer | dust | Control locations | |  |
| *A. tubingensis* | MFBF AN12136 | Winter | dust | Repaired locations | |  |
| *A. tubingensis* | MFBF AN12138 | Winter | dust | Repaired locations | |  |
| *A. tubingensis* | MFBF AN12140 | Winter | dust | Repaired locations | |  |
| *A. tubingensis* | MFBF AN12400 | Summer | dust | Repaired locations | |  |
| *A. tubingensis* | MFBF AN12512 | Winter | dust | Repaired locations | |  |
| *A. tubingensis* | MFBF AN12513A | Winter | dust | Repaired locations | |  |
| *A. tubingensis* | MFBF AN12685 | Summer | dust | Repaired locations | |  |
| *A. tubingensis* | MFBF AN12690 | Summer | dust | Repaired locations | |  |
| *A. tubingensis* | MFBF AN12403B | Summer | dust | Unrepaired locations | |  |
| *A. tubingensis* | MFBF AN12506 | Winter | dust | Unrepaired locations | |  |
| *A. uvarum* | MFBF AN12686 | Summer | dust | Repaired locations | |  |
| *A. welwitschiae* | MFBF AN12208 | Winter | dust | Control locations | | 43.84 |
| *A. welwitschiae* | MFBF AN12210 | Winter | dust | Control locations | |  |
| *A. welwitschiae* | MFBF AN12412A | Summer | dust | Control locations | |  |
| *A. welwitschiae* | MFBF AN12412B | Summer | dust | Control locations | |  |
| *A. welwitschiae* | MFBF AN12413 | Summer | dust | Control locations | |  |
| *A. welwitschiae* | MFBF AN12564 | Winter | dust | Control locations | | 8.01 |
| *A. welwitschiae* | MFBF AN12565A | Winter | dust | Control locations | | 10.07 |
| *A. welwitschiae* | MFBF AN12565B | Winter | dust | Control locations | | 0.51 |
| *A. welwitschiae* | MFBF AN12566 | Winter | dust | Control locations | |  |
| *A. welwitschiae* | MFBF AN12567A | Winter | dust | Control locations | |  |
| *A. welwitschiae* | MFBF AN12567B | Winter | dust | Control locations | |  |
| *A. welwitschiae* | MFBF AN12742A | Summer | dust | Control locations | | 3.2 |
| *A. welwitschiae* | MFBF AN12742B | Summer | dust | Control locations | | 0.19 |
| *A. welwitschiae* | MFBF AN12744A | Summer | dust | Control locations | |  |
| *A. welwitschiae* | MFBF AN12744B | Summer | dust | Control locations | |  |
| *A. welwitschiae* | MFBF AN12399 | Summer | dust | Repaired locations | |  |
| *A. welwitschiae* | MFBF AN12511 | Winter | dust | Repaired locations | |  |
| *A. welwitschiae* | MFBF AN12513B | Winter | dust | Repaired locations | |  |
| *A. welwitschiae* | MFBF AN12513C | Winter | dust | Repaired locations | |  |
| *A. welwitschiae* | MFBF AN12513D | Winter | dust | Repaired locations | |  |
| *A. welwitschiae* | MFBF AN12687 | Summer | dust | Repaired locations | | 0.04 |
| *A. welwitschiae* | MFBF AN12689 | Summer | dust | Repaired locations | |  |
| *A. welwitschiae* | MFBF AN12405 | Summer | dust | Unrepaired locations | |  |
| *A. welwitschiae* | MFBF AN12406 | Summer | dust | Unrepaired locations | |  |
| *A. welwitschiae* | MFBF AN12505 | Winter | dust | Unrepaired locations | |  |
| *A. welwitschiae* | MFBF AN12693 | Summer | dust | Unrepaired locations | | 0.19 |
|  |  | N producing dust-borne isolates/N total dust-borne isolates | | | | 9/45 |
|  |  |  | | | |  |
| FB_2_ producing species | | N producing isolates/N total isolates | | | Concentration (µg/ml) arithmetic mean ± SD | |
| *A. niger* | | 3/5 | | | 11.24 ± 18.30 | |
| *A. welwitschiae* | | 10/41 | | | 6.76 ± 13.51 | |
