# Supplementary figures and images for: Post-Flood Impacts on Occurrence and Distribution of Mycotoxin-Producing Aspergilli from the Sections Circumdati, Flavi, and Nigri in Indoor Environment
Source: J Fungi (Basel). 2020 Nov 12;6(4):282. doi: 10.3390/jof6040282 (PMC7711759; doi:10.3390/jof6040282)

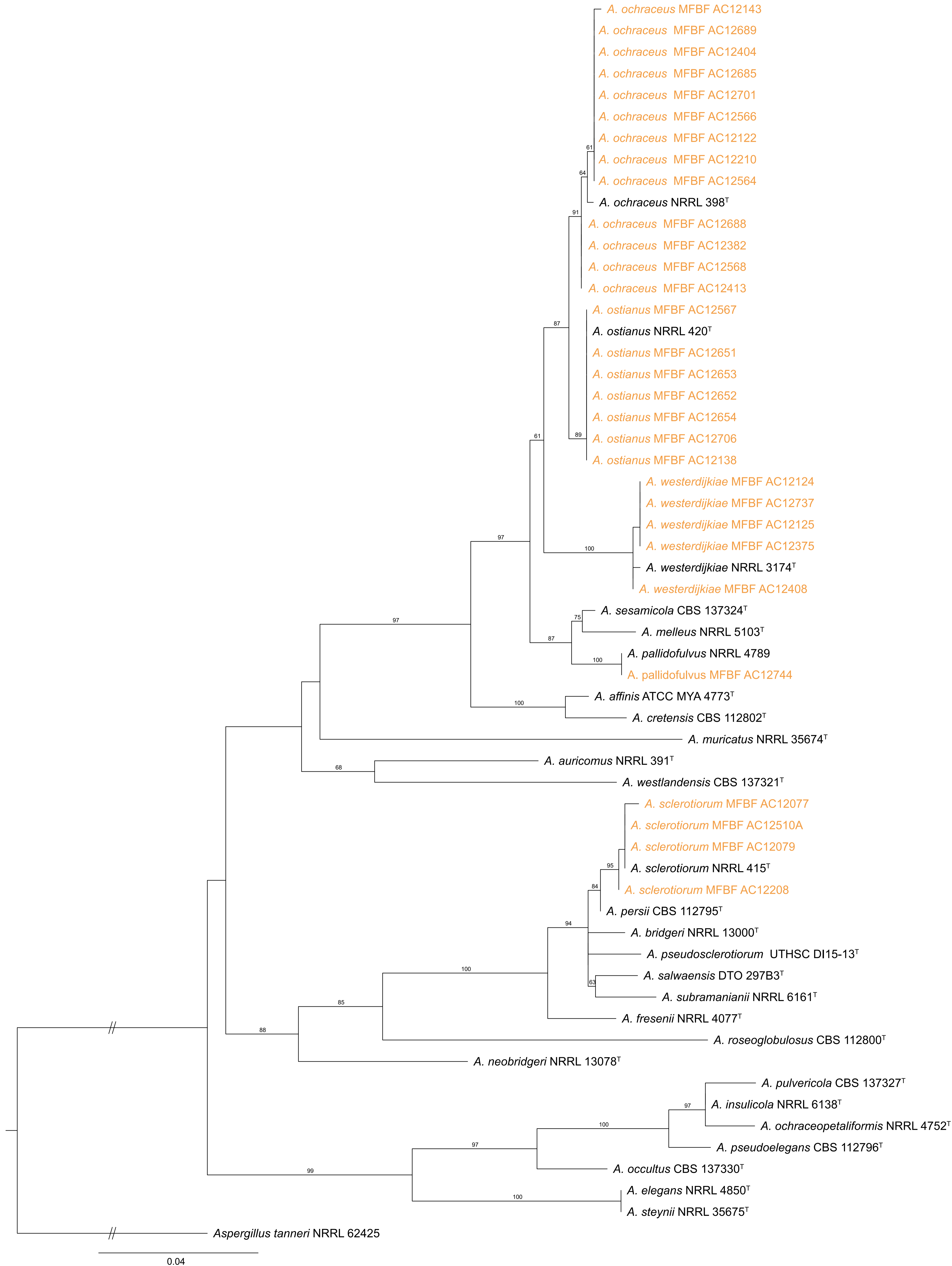

Supplement: Supplementary file 1 [file jof-06-00282-s001.zip › Supplementary Materials/Figure S1.pdf]

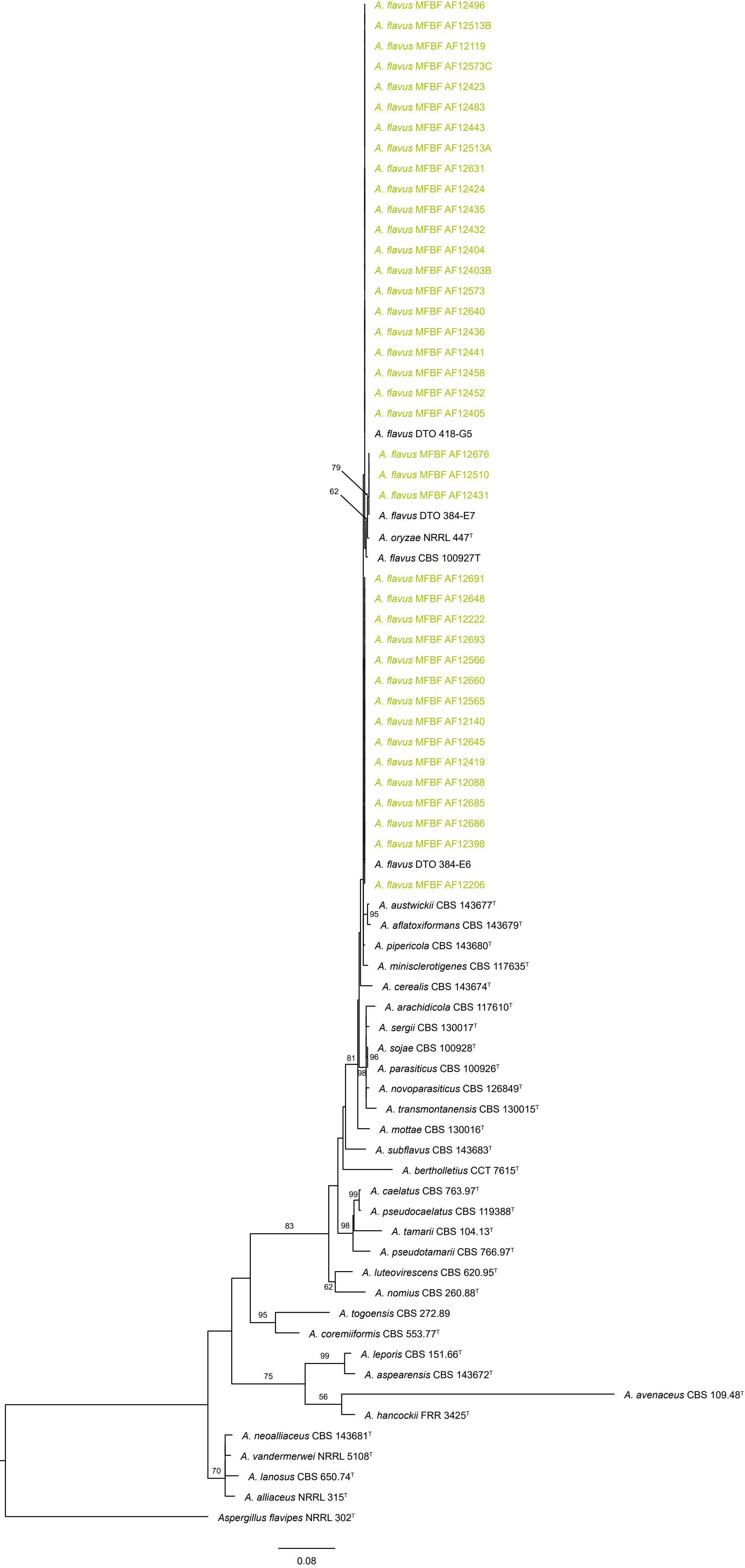

Supplement: Supplementary file 1 [file jof-06-00282-s001.zip › Supplementary Materials/Figure S2.pdf]

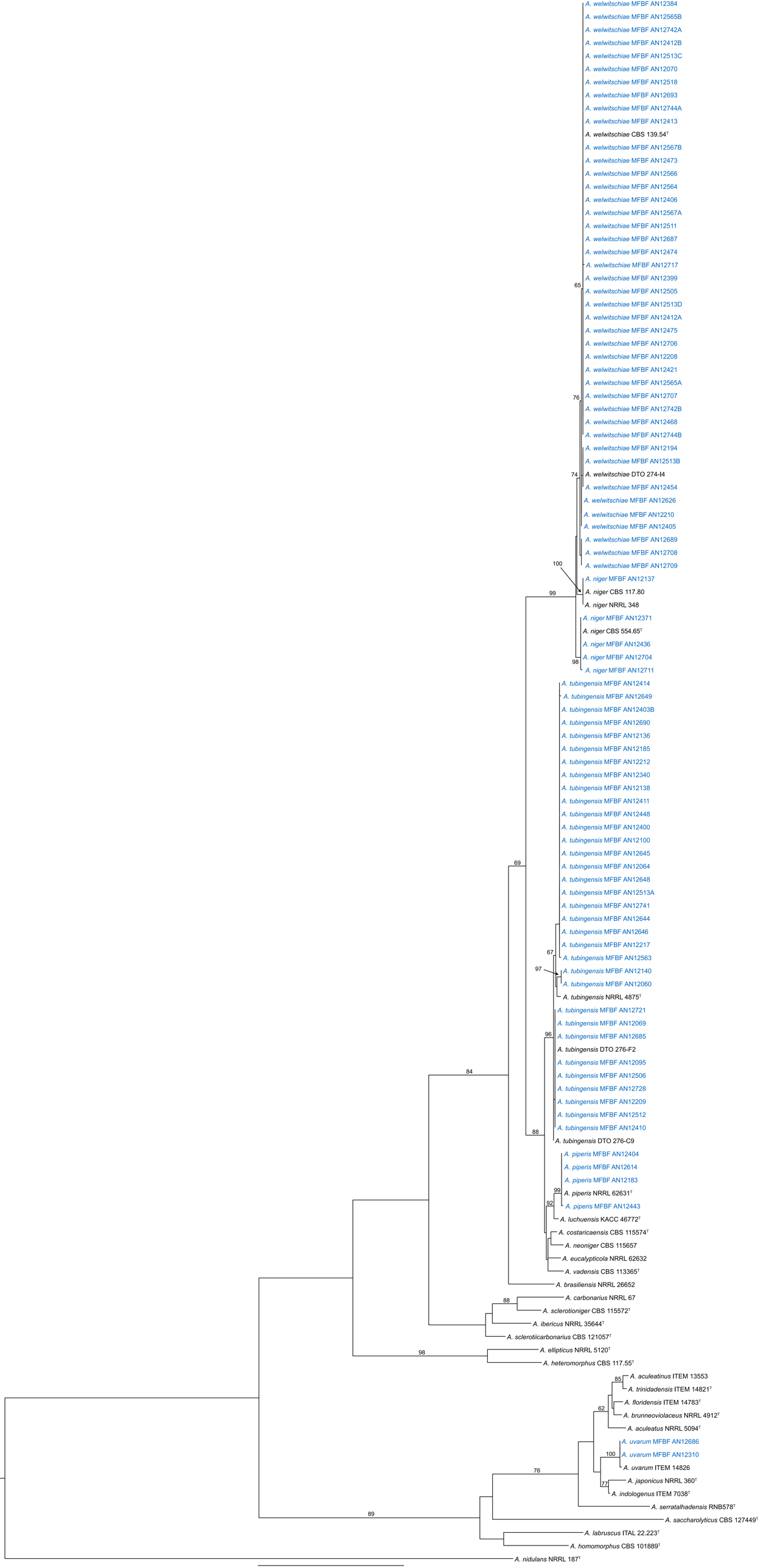

Supplement: Supplementary file 1 [file jof-06-00282-s001.zip › Supplementary Materials/Figure S3.pdf]
